# In Situ Synthesis of Porous SnO_2_/SnS_2_@PC Anode Material with High Capacity Using Calcium Carbonate as Template for Lithium-Ion Batteries

**DOI:** 10.3390/ma18214987

**Published:** 2025-10-31

**Authors:** Wen Chen, Chunling Li, Mengyang Zheng, Yanlin Li, Fuzhong Gong

**Affiliations:** 1 Guangxi Key Laboratory of Special Non-Wood Forests Cultivation and Utilization, Nanning 530002, China; chenwen_gfri@163.com (W.C.);; 2 Guangxi Laboratory of Foresty, Guangxi Forestry Research Institute, Nanning 530002, China; 3 College of Chemistry and Chemical Engineering, Guangxi University, Nanning 530004, China

**Keywords:** Tin-based anode, petroleum asphalt, porous carbon, calcium carbon, lithium-ion batteries

## Abstract

Tin-based materials have emerged as promising anode candidates for advanced lithium-ion batteries (LIBs) due to their high theoretical capacity (e.g., 994 mAh·g^−1^ for Li_4_._4_Sn), moderate operating potential, and natural abundance. However, Tin-based materials suffer from severe volume expansion (>300%) and rapid capacity decay during cycling. To mitigate these challenges, a composite composed of tin-based materials and porous carbon (PC), i.e., SnO_2_/SnS_2_@PC, was prepared by calcining a mixture of SnO_2_, petroleum asphalt and calcium carbonate at high temperature, where petroleum asphalt acted as the carbon and sulfur resource, and calcium carbonate acted as a pore-forming template. The prepared SnO_2_/SnS_2_@PC composite had a specific surface area of 190 m^2^·g^−1^ with total pore volume 0.386 cm^3^·g^−1^, and delivered an initial specific capacity of 1431 mAh·g^−1^ and retained 722 mAh·g^−1^ at 100th cycle at 0.2 A·g^−1^, which is nearly three folds that of the actual capacity (~260 mAh·g^−1^) of commercial graphite. The novelty of this work lies in that the abundant sulfur element in petroleum asphalt was fully utilized to react in situ with nano SnO_2_ to generate SnS_2_ and form a composite with high specific capacity and good structural stability, along with greatly reducing the emission of the harmful element sulfur into the atmosphere.

## 1. Introduction

Nowadays, lithium-ion batteries (LIBs) are the preferred power source for electric- vehicles, grid storage, and portable electronic devices due to their large voltage window, high energy density and conversion efficiencies, reasonable life spans, and safety [1,2]. For consumers, especially those who own electric vehicles, the duration of continuous use after a single charge is a matter of great concern. This directly depends on the capacity of a battery, which is determined by the specific capacity of the electrode materials used in the battery (includes cathode material and anode material). With regard to the anode material of LIBs, graphite remains the commercially dominant choice, owing to its excellent conductivity, stable layered structure, low cost, and mature industrialization technology, but it cannot meet the criteria for emerging high-end applications in commercial markets because of its inferior theoretical specific capacity (372 mAh·g^−1^). Furthermore, the dendritic lithium deposition at low potential over graphite can decline the performance and affect safety issues [3,4].

To develop alternative anode materials with higher capacity, over the last decade or so, the graphite-alternative anode materials such as silicon-based materials [5], black phosphorus [6], transition metal oxides and sulfides [7,8,9,10,11,12,13,14,15], and lithium titanite oxide (LTO) [16] have attracted widespread attention due to their higher specific capacity or superior rate performance. Among these candidate materials, Tin-based materials are deemed as the most potential alternatives owing to their low voltage, high theoretical specific capacity (e.g., 994 mAh/g for Li_4_._4_Sn), and relative abundance on Earth. However, there are several obstacles restricting their wholesale applications, such as the severe specific volume variation in conversion-reaction during the charge/discharge process, which results in structural instability (pulverization), rapid capacity decline at high-rate conditions, and poor electrical conductivity [17]. To solve these issues, the integration of Tin-based materials with specific structural carbon is an effective approach. The commonly applied carbonaceous materials are graphite, graphene, carbon nanotubes (CNTs), and carbon nanofibers (CNFs) [18,19]. The incorporated carbon materials have the ability to improve the electrical conductivity, reduce the particle agglomeration, and effectively restrain the volume expansion of the particles due to the layered or porous structure, which provides a buffer space for volume expansion, thereby improving the electrochemical performance of the hybrid materials. For example, Choi et al. synthesized a SnO_2_/nano-perforated graphene composite by conversion reaction of Sn and Li_2_O to SnO_2_, and displayed an initial capacity of 1446 mAh·g^−1^ at the current density of 100 mAh·g^−1^ [8]. Li et al. fabricated a SnO_2_/SnS_2_@carbon/N-doped (SnO_2_-SnS_2_@C/NG) composite by using SnCl_4_, sodium alginate, reduced graphene, and thioacetamide as start materials; this composite showed a high initial capacity of 1201.2 mAh·g^−1^ at the current density of 100 mA·g^−1^ [9]. Syum et al. prepared a SnS_2_-CNT-CC anode material with a capacity of 645 mAh·g^−1^ after 100 cycles [10]. Cheng et al. reported an SnS_2_/CNTs composite, which showed a capacity of 660 mAh·g^−1^ after 100 cycles [11]. Jin et al. synthesized a SnS_2_/SnO_2_@C/rGO nanocomposite by using SnCl_2_, thiourea, L-ascorbic acid, and graphene oxide (GO) as reactants, which exhibited a reversible capacity of 689 mAh·g^−1^ at a current density of 78.3 mA·g^−1^ [12]. These Tin-based hybrids show a significant improvement in capacity compared to graphite, but all these materials utilized expensive graphene or CNTs.

Porous carbon has the advantage of high conductivity, stability, surface area, and low cost, which can be prepared via in situ templating synthesis [20]. The cheap carbon sources for preparing porous carbon mainly include bitumen (petroleum bitumen and coal tar pitch) and biomass [21,22,23]. Some thermally unstable chemical compounds such as citrates, oxalates, acetates, and basic carbonates are the most widely used templates [24]. The extremely cheap calcium carbonate (CaCO_3_) also can be used as a pore-forming template, because it decomposes to calcium oxide (CaO) and carbon dioxide (CO_2_) at over 700 °C [25]. Although the decomposition temperature is higher, it is conducive to the graphitization of carbon, which is essential for porous carbon as an anode material.

Petroleum asphalt is a very abundant and cheap carbon material for producing porous carbon; it is a black–brown mixture of polycyclic aromatic hydrocarbons of different molecular weights and non-metallic derivatives [21]. Petroleum asphalt also contains a certain amount of sulfides, which may be oxidized to produce sulfur dioxide, a harmful air pollutant that can form acid rain. In this study, we utilized petroleum asphalt as a carbon and sulfur source, CaCO_3_ as the pore-forming template, and SnCl_4_ as starting Tin source to synthesize in situ a porous SnO_2_/SnS_2_@PC anode material through high-temperature carbonization. This as-prepared SnO_2_/SnS_2_@PC anode delivered an initial specific capacity of 1431 mAh·g^−1^ at 0.2 A·g^−1^ and retained ~722 mAh·g^−1^ at 100th cycle, which is nearly three folds that of the actual capacity (~260 mAh·g^−1^) of commercial graphite, and maintains good structural stability during the charge and discharge processes. The strategy proposed in this article not only successfully fabricated a Tin-based anode material of LIBs with significantly improved specific capacity, but also greatly reduced sulfur emissions from asphalt, hence exhibiting promising application prospects.

## 2. Materials and Methods

### 2.1. Chemicals

Petroleum asphalt was purchased from China Petroleum & Chemical Corporation, Beijing, China. The CaCO_3_ with a spherical shape and ~3 μm in size was synthesized by our group through the reaction of Na_2_CO_3_ (0.1 M) with CaCl_2_ (0.1 M). All other reagents were analytical grade and used without further purification. Ultrapure water was obtained through reverse osmosis membranes (electrical resistivity is 18.2 MΩ·cm) and was used for making all aqua solutions.

### 2.2. Synthesis of SnO_2_/SnS_2_@PC Composite

**Figure 1:**
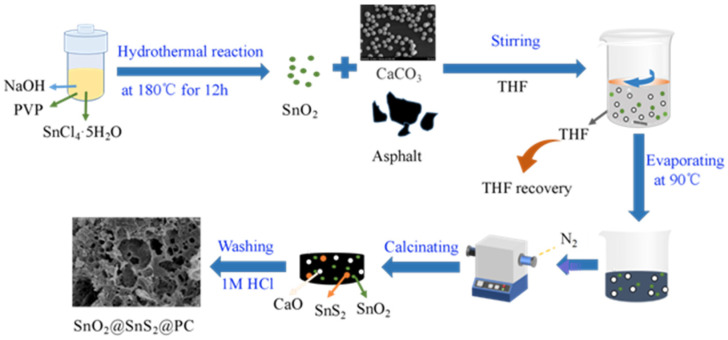
The schematic preparation procedure of SnO_2_/SnS_2_@PC composite.

(1) Synthesis of nano-SnO_2_. The nano-SnO_2_ was synthesized by a hydrothermal method. In brief, 1.40 g SnCl_4_·5H_2_O, 0.1.20 g NaOH, and 0.3 g PVP were dissolved in a 30 mL ethanol–water (1:1) solution and continuously stirred magnetically until transparent. Next, the solution was transferred into a 100 mL Teflon-lined stainless-steel autoclave, which was then placed in an oven and heated at 180 °C for 12 h. After cooling to room temperature, the final product (nano-SnO_2_) was centrifuged and washed three times with water. After drying under vacuum at 80 °C for 12 h, the obtained white powder was fully ground and stored for later use.

(2) Synthesis of SnO_2_/SnS_2_@PC composite. First, 50 mL of tetrahydrofuran (THF) and 7.5 g of asphalt were added sequentially into a 100 mL beaker and stirred until completely dissolved. Then, 0.5 g of SnO_2_ and 2 g of CaCO_3_ were added to the solution and stirred continuously for 30 min. Next, the mixture was transferred to a rotary evaporator and heated in a water bath at 90 °C to recover the THF. We transferred the resulting residue to a ceramic crucible, placed it in a tube furnace, and calcinated at 900 °C for 2 h under a nitrogen atmosphere. After natural cooling, the calcined residue was alternately washed with 1 M HCl solution and pure water three times to remove the CaO produced by the decomposition of CaCO_3_, and finally, it was dried in a vacuum oven at 80 °C. The obtained product was denoted as SnO_2_/SnS_2_@PC, where the SnS_2_ was formed by the combination of sulfur elements in asphalt and Sn in SnO_2_, which can be confirmed by the analysis results of XRD and XPS. For comparison, the SnO_2_/SnS_2_@C and pure carbon samples were prepared in the absence of CaCO_3_ or SnO_2_ and CaCO_3_ under the same conditions, respectively. The schematic procedure preparation of SnO_2_/SnS_2_@PC is illustrated in Figure 1.

### 2.3. Characterization

The SEM and TEM observations were performed by TESCAN MIRA LMS (TESCAN ORSAY HOLDING, a.s., Brno, Czech) and Tecnai G2 F30 (Thermo Fisher Scientific, Hillsboro, OR, USA), respectively. The Powder X-ray diffraction (XRD) analysis was carried out in an X-ray diffractometer (Bruke D8, Bruker Corporation, Karlsruhe, Germany) with Cu Kα radiation (λ = 0.1540 nm) at 2θ from 10 to 90°. The X-ray photoelectron spectroscopy (XPS) was conducted on a Thermo Scientific K-Alpha spectrometer (Thermo Fisher Scientific, East Grinstead, UK) using C 1s (284.8 eV) as a reference. Raman spectra were recorded on a Raman spectrometer (WIT ecalpha300R, WITec Wissenschaftliche Instrumente und Technologie GmbH, UIm, Germany) with an excitation wavelength of 532 nm. Nitrogen adsorption/desorption isotherms were recorded on a Micromeritics instrument (ASAP 2460, Micromeritics Instrument Corporation, Norcross, GA, USA).

### 2.4. Electrochemical Measurements

All electrochemical tests were conducted using a CR2025 coin-type cell, in which the working electrode and anode were the as-synthesized active material and lithium foil, respectively, and a polypropylene microporous membrane (Celgand 2400) was used as a separator. The electrolyte was 1 M LiPF6 dissolved in EC/DMC/EMC (1:1:1, *v*/*v*/*v*). The preparation of the working electrode included the following steps: 0.8 g active material, 0.1 g acetylene black, and 0.1 g polyvinylidene fluoride (PVDF) were mixed with 5 mL of 1-methyl pyrrolidone (NMP) and magnetically stirred for 6 h to form a slurry. The slurry was then coated on Cu foil and dried in a vacuum oven at 80 °C for 8 h. After that, the copper foil was punched into discs with a diameter of 14.0 mm, on which the average active materials loading was about 1.1 ± 0.05 mg cm^−2^. The assembled CR2025 cells were assembled in an argon-filled glove box and tested after standing for 8 h. The galvanostatic charge/discharge tests were carried out in a battery measurement system (LAND CT2001A, Wuhan LAND Electronic Co., Ltd., Wuhan, China) in a 0.01–3.0 V voltage range vs. Li^+^/Li. Cyclic voltammetry (CV) and electrochemical impedance spectroscopy (EIS) tests were performed in an electrochemical working station (CHI660E, Shanghai Chenhua Instrument Co., Ltd., Shanghai, China). The CV curves were monitored at a scanning rate of 0.1 mV·s^−1^ within a voltage 100 kHz with an AC potential amplitude of 5 mV. All electrochemical measurements were conducted at 25 ± 1 °C.

## 3. Results

**Figure 2:**
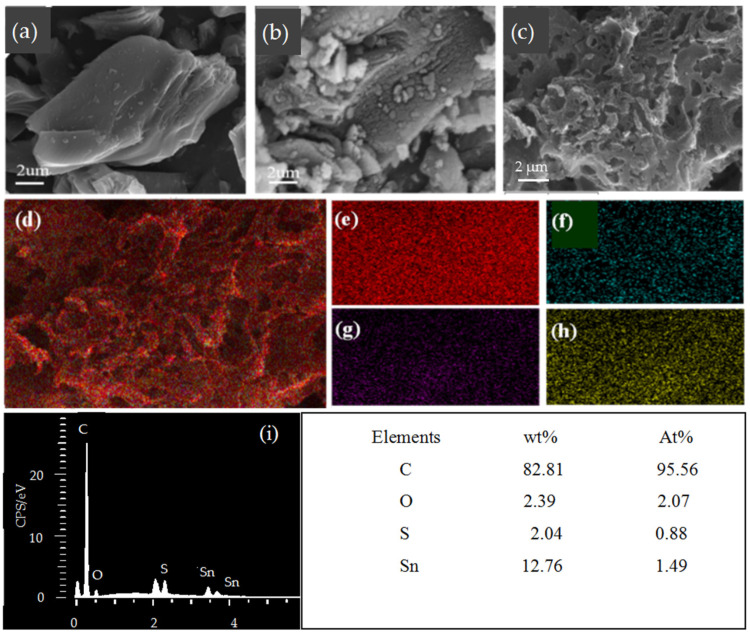
SEM images of (**a**) pure C; (**b**) SnO_2_/SnS_2_@C; (**c**) SnO_2_/SnS_2_@PC; (**d**–**h**) maps of total elements distribution and C, O, S, Sn elements; (**i**) element contents of SnO_2_/SnS_2_@PC.

Figure 2a–c show the SEM images of the prepared Pure-C, SnO_2_/SnS_2_@C, and SnO_2_/SnS_2_@PC composites. As displayed in Figure 2a,b, the particle morphology of Pure-C and SnO_2_/SnS_2_@C presented layered structure and no pores were observed—some nano particles (SnO_2_ and SnS_2_) were attached to the surface of the carbon matrix. Different from these two composites, the SnO_2_/SnS_2_@PC composite possess a three-dimensional (3D) porous structure which was constructed from carbon nanosheets with many open mesopores of 30~200 nm. As depicted in Figure 2c, the large pores can be reasonably inferred to have been formed by the escape of CO_2_ gas, and the small pores were formed by the dissolve of CaO existing within in the composite. Both CO_2_ and CaO were the decomposition products of CaCO_3_ at high temperature. Additionally, the elemental distribution and content of C, O, S, and Sn in SnO_2_/SnS_2_@PC was probed by energy-dispersive X-ray spectroscopy (EDS). The mapping images shown in Figure 2d–h reveal that the C, O, S, and Sn elements were evenly scattered all over the carbon matrix, and the molar ratio of SnO_2_ to SnS_2_ is estimated to be 7/3 according to the atom percentage of O, S, and Sn as shown in the inset table of Figure 2i. The nanonization of SnO_2_ and SnS_2_ and their uniform distribution within the matrix are crucial for the electrochemical performance and structural stability of the material. This nanonization and uniform distribution stem from the following two reasons. Firstly, sulfur-containing functional groups in asphalt (particularly those with strong polarity) can serve as coordination or adsorption sites for Sn ions. This interaction enables the uniform dispersion of SnO_2_ within the asphalt, preventing aggregation into large particles and ultimately resulting in uniformly distributed SnS_2_. Moreover, compared with physically mixed SnO_2_/SnS_2_, the in situ formation of SnS_2_ nano particles were more evenly distributed in the composite material, thus having better electrochemical performance. Secondly, during the high-temperature carbonization process, sulfur elements in petroleum asphalt generate active sulfur radicals (S^2−^), which firstly diffuse into SnO_2_, while the O^2−^ and Sn ions on tin precursors (SnO_2_) migrate outward and react with S^2−^ to form SnS_2_. The smaller the SnO_2_ particle, the lower the resistance to this solid-state phase transition process, the faster the rate of SnO_2_ → SnS_2_ conversion. Small sized particles of SnO_2_ and SnS_2_ typically enable more efficient electrochemical reactions, as more active species (especially internal atoms) can be utilized, resulting in higher reversible capacity.

**Figure 3:**
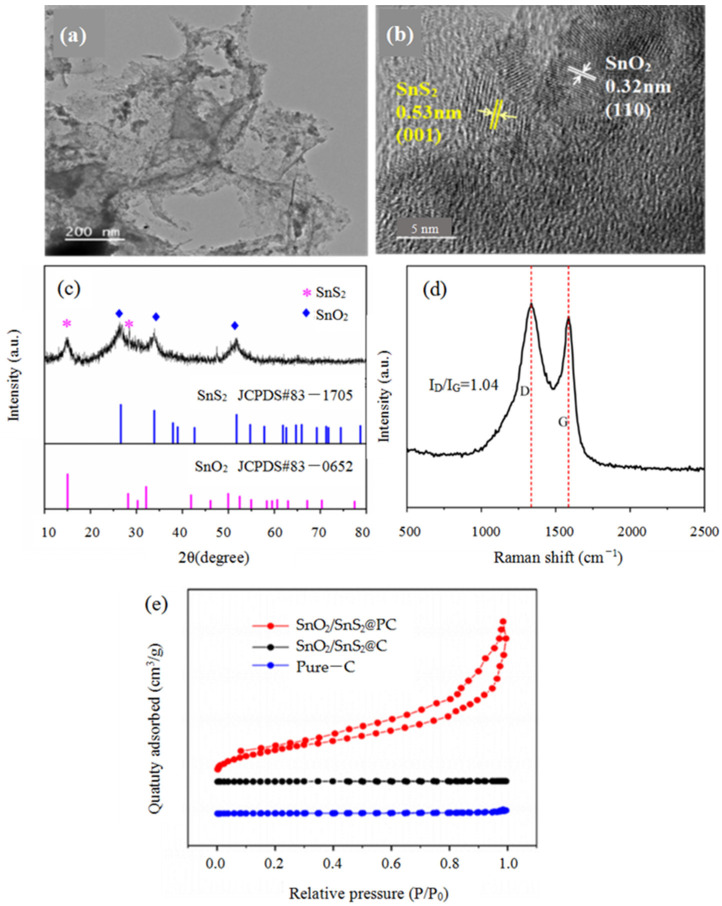
(**a**) TEM image of SnO_2_/SnS_2_@PC; (**b**) HRTEM image of SnO_2_/SnS_2_@PC; (**c**) XRD patterns of SnO_2_/SnS_2_; (**d**) Raman spectrum; (**e**) N_2_ adsorption isotherms.

The TEM image (Figure 3a) of SnO_2_/SnS_2_@PC shows that many extremely tiny SnO_2_ and SnS_2_ nano-spherical particles are incorporated with the porous sheet-like carbon skeleton matrix. The HR-TEM image (Figure 3b) indicates the lattice stripes of (110) crystal planes of SnO_2_ and (100) crystal planes of SnS_2_ are 0.32 nm and 0.53 nm, respectively. The XRD pattern of the SnO_2_/SnS_2_@PC composite is illustrated in Figure 3c, where the main characteristic peaks of SnO_2_ and SnS_2_ simultaneously appear, confirming that both SnO_2_ and SnS_2_ are presented in the composite, where the SnS_2_ was conversed by reaction of partial SnO_2_ with the S element existing in asphalt during the high-temperature calcination process. The strongest peak at 26.6° (110) and two other major diffraction peaks at 33.9° (101) and 51.7° (211) can be clearly seen, corresponding to the tetragonal phase of SnO_2_ (JCPDS 71-0652) with a spatial group of P42/mnm. The strongest characteristic peak at 15.1° aligns with the crystallographic diffraction peak of SnS_2_ of the (001), which is the primary basis for determining its existence, and the peaks are at 28.2°, 32.1° index to (100) and (101) crystal planes, matching the hexagonal phase of SnS_2_ (JCPDS 83-1705) with a spatial group of P-3m1 [8,9,10,11].

The Raman spectroscopy was conducted to characterize the structure of carbon material in the SnO_2_/SnS_2_@PC composite. As shown in Figure 3d, both the D-band located at 1350 cm^−1^ and G-band located at 1580 cm^−1^ are clearly visible, the appearance of the D-band derived from the carbon six-membered ring indicates that there are defects, disorders, or boundaries in the graphite lattice, while the G-band caused by the in-plane stretching vibration of the sp2 hybrid orbitals of carbon atoms reveals the existence of an ordered graphitized structure in the sample [26,27]. The degree of defects in the porous carbon can be evaluated by the intensity ratio (I_D_/I_G_), which is calculated to be approximately 1.04, suggesting that the degree of defects is slightly higher than that of the degree of graphitization of the prepared porous carbon. The textural properties of the three samples were studied using nitrogen adsorption/desorption isotherm analyses as presented in Figure 3e. The isotherm of SnO_2_/SnS_2_@PC consists of an apparent hysteresis (H4) loop, which was detected within the relative pressure (P/P_0_) region of ~0.3 to 1.0, vesting in type IV gas sorption characteristics according to the IUPAC classification, reflecting their mesoporous structure [23,28]. Moreover, the N_2_ adsorbed quantity increases sharply at relatively high pressure (P/P_0_ close to 1), which is caused by large pores [29,30]; this is in accordance with the SEM observation shown in Figure 2a,b. For comparison, the nitrogen adsorption isotherms of pure C and SnO_2_/SnS_2_@C display very limited nitrogen adsorption capacity, confirming their non-porous structure. The specific surface area (S_Total_) and total pore volume (V_Total_) are calculated as 190 m^2^·g^−1^ (Brunauer–Emmett–Teller, BET) and 0.386 cm^3^·g^−1^ (Barrett–Joyner–Halenda, BJH), respectively. Both the specific surface area and pore volume are superior to those of Pure-C (2.3 m^2^·g^−1^, 0.001 cm^3^·g^−1^) and SnO_2_/SnS_2_@C (3 m^2^·g^−1^, 0.005 cm^3^·g^−1^). The significant increases in specific surface area and total pore volume for SnO_2_/SnS_2_@PC attribute to the results of CO_2_ escaping and CaO being dissolved by hydrochloric acid to form a porous structure. Both CO_2_ and CaO are products of the decomposition of CaCO_3_ at high temperatures. The specific surface area and pore structure parameters are accordingly calculated and listed in Table 1. By comparing the specific surface areas of micropores (S_Mic_), mesopores (S_Mec_), pore volumes of macropores (V_Mac_), and mesopores (V_Mec_), we can draw the conclusion that the presence of mesopores makes the predominant contribution to the total surface area and total pore volume.

As has been noted above, the main problem of Sn-based anode materials is their huge volume expansion (~300%) during the cyclic charging/discharging process, which leads to structural damage and poor cycle life. The high porosity carbon matrix provides a critical physical buffer space for volume expansion and is the physical basis for preventing electrode structure collapse. Meanwhile, the carbon matrix with a high specific surface area enhances the contact between Sn and carbon and improves the conductivity and mechanical constraint. However, too high of a specific surface area will have more extensive contact with electrolytes, resulting in more SEI film forming on the carbon surface, which consumes a large amount of lithium ions and electrolytes, resulting in low initial Coulombic efficiency and rapid capacity decay. In practical applications, porosity and specific surface area are interrelated and need to be considered synergistically. The synthesized SnO_2_/SnS_2_@PC composite is mainly composed of mesopores, which can provide sufficient buffer space while avoiding excessive specific surface area (extremely small proportion of micropores), making it have good buffering volume expansion ability and long-term structural stability.

**Figure 4:**
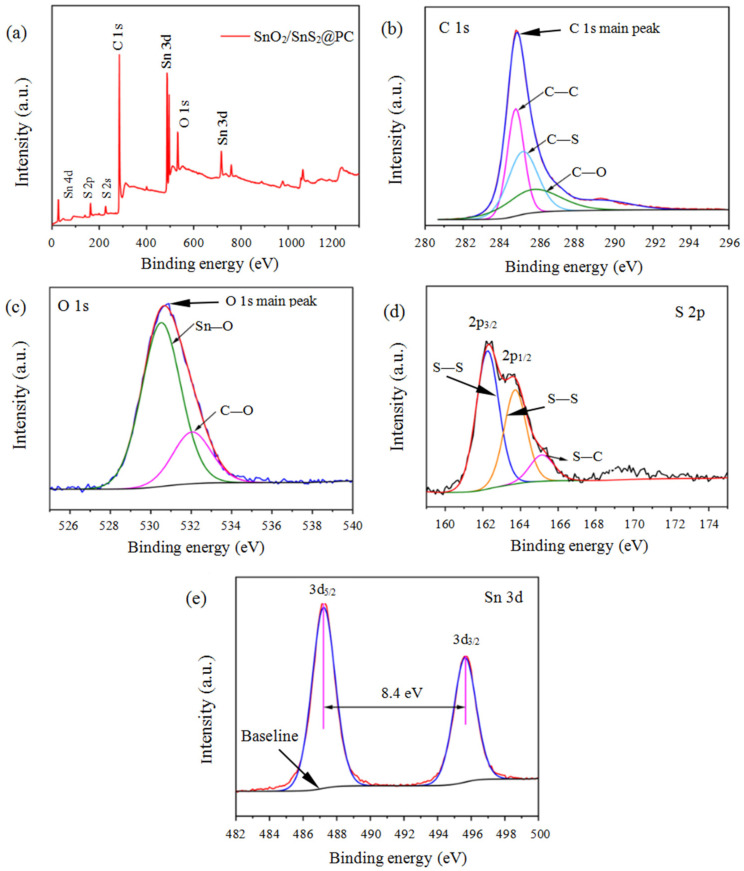
(**a**) Survey XPS spectrum of SnO_2_/SnS_2_@PC; (**b**–**e**) high-resolution XPS spectra of C 1S, O 1S, S 2P, and Sn 3d; (**c**) XRD patterns of SnO_2_/SnS_2_; (**d**) Raman spectrum; (**e**) N_2_ adsorption isotherms.

The chemical composition and electronic state of the composite surface were analyzed by XPS. Figure 4a displays the survey spectrum of the SnO_2_/SnS_2_@PC composite, confirming the presence of four elements of C, O, S, and Sn. Figure 4b–e display the high-resolution XPS spectra of C 1s, O1s, S 2p, and Sn 3d. The peaks located at a binding energy of 284.8, 285.2, and 286.1 eV in C 1s spectrum (Figure 4b) are attributed to C−C, C−S, and C−O, respectively [31]. The peaks at the energy of 530.5 eV and 532 eV in the O 1s spectrum (Figure 4c) are ascribed to Sn−O−Sn and C−O−Sn bonds, respectively, and validates the valence state of O as −2 [32,33]. Figure 4d represents the deconvoluted S 2p spectrum, where the peak located at a binding energy of 162.1 eV (S 2P_3/2_) assigns to the S–S bond, the peak at 163.3 eV (S 2P_3/2_) is specified metal-S bonding, and the peak located at 164.9 eV (S 2P_1/2_) belongs to the S-C covalent bond [34,35]. The two peaks located at a binding energy of 487.2 eV and 495.6 eV (Figure 4e) belong to Sn 3 d_5/2_ and Sn 3 d_3/2_, respectively, and the splitting energy between Sn 3d_5/2_ and Sn 3d_3/2_ is 8.4 eV. Such a Sn 3d spectrum designates the Sn^4+^ state [36]. The results of the XPS analysis demonstrate that both SnO_2_ and SnS_2_ were present in the SnO_2_/SnS_2_@PC composite, which is consistent with the XRD analysis results.

### Electrochemical Properties of SnO_2_/SnS_2_@PC Composite

**Figure 5:**
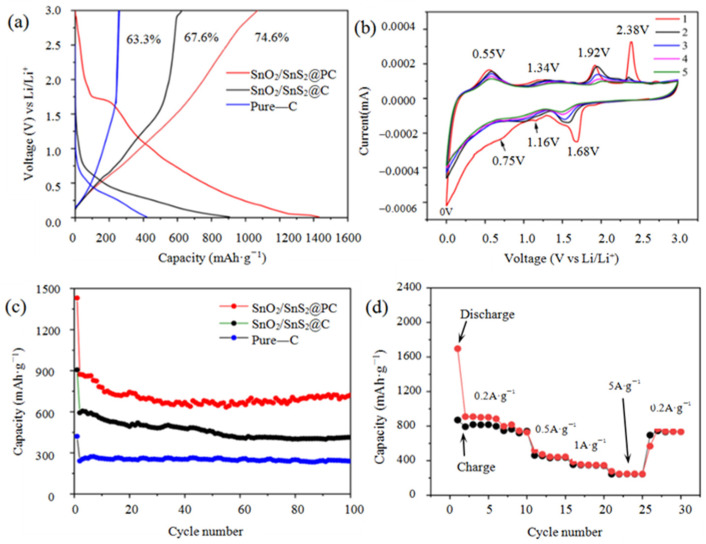
(**a**) Constant current charge/discharge curves in first cycle at 0.2 A·g^−1^; (**b**) CV curves within 1~5 cycles at 0.1 mv/s of SnO_2_/SnS_2_@PC; (**c**) cyclic performance within 100 cycles; (**d**) rate performance at 0.2~5.0 A·g^−1^ of SnO_2_/SnS_2_@PC.

The Li-ion storage outcomes of Pure-C, SnO_2_/SnS_2_@C, and SnO_2_/SnS_2_@PC as LIB anodes were evaluated using the CR-2025 coin cell assembly vs. Li metal. Figure 5a presents the galvanostatic charge/discharge (GCD) profiles for the first cycle captured at a current density of 0.2 A·g^−1^. As is shown, the initial charge/discharge specific capacity were determined to be 1068/1431 mAh·g^−1^ (SnO_2_/SnS_2_@PC), 613/906 mAh·g^−1^ (SnO_2_/SnS_2_@C), and 266/421 mAh·g^−1^ (Pure-C), with the initial Coulomb efficiency (CE) of 74.6%, 67.7%, and 63.2%, respectively. The reason for the relative low efficiency of the first cycle is as follows: during the first charging process, the electrolyte will undergo electrochemical reduction decomposition on the surface of the anode material, forming a passive layer (SEI film) covering the anode surface. The formation of SEI film requires the consumption of lithium salts and solvent molecules in the electrolyte and permanently immobilizes a portion of lithium ions in these decomposition products. This fixed lithium cannot return to the cathode during discharge, resulting in direct and permanent capacity loss. In this study, the Coulomb efficiency follows the following evolution pattern during cycles as follows: in the first cycle, the Coulomb efficiency was only 74.6%, and in the subsequent second to tenth cycles, the Coulomb efficiency rapidly increased. This is because after the formation of the SEI film in the first lap, the main irreversible reactions have already been completed. After ten cycles, the Coulomb efficiency gradually stabilized and approached 100% infinitely, indicating that the chemical reactions inside the battery tended to stabilize, and the reversible lithium ion insertion/extraction process became dominant.

The porous SnO_2_/SnS_2_@PC anode exhibited the highest specific capacity for the first cycle, which is about four times than that of Pure-C. The non-porous SnO_2_/SnS_2_@C anode also showed a significant improvement over carbon, being more than twice as good as Pure-C. This outcome stems from the reaction between Li^+^ ions and SnO_2_, as well as SnS_2_ and Sn during the charge/discharge process as described by the following Equations (1)–(3) [37,38]:

SnO_2_ + 4Li^+^ + 4e^−^ → Sn + 2Li_2_O (1)

SnS_2_ + 4Li^+^ + 4e^−^ → Sn + 2Li_2_S (2)

Sn + xLi^+^ + xe^−^ → Li_x_Sn (0 < x < 4.4) (3)

Aside from this, it is clear that the specific capacity of non-porous SnO_2_/SnS_2_@C is lower than that of porous SnO_2_/SnS_2_@PC, which can be attributed to the fact that the SnO_2_ and SnS_2_ nanoparticles in the latter were able to be more exposed and participated in the redox reactions during the charging/discharging process. Figure 5b shows the cyclic voltammetry (CV) curves of SnO_2_/SnS_2_@PC electrode for the first five cycles monitored at a scan rate of 0.1 mV s^−1^ within a fixed potential domain (0.01–3.0 V). In the first cathodic scan, one prominent peak (~1.68 V) and two weak peaks (~0.75 and 1.16 V) are observed. The peak at ~0.75 V indicates the formation of the solid electrolyte interface (SEI) film on the electrode surface, which is ascribed to the decomposition of the electrolyte during initial Li-ions intercalation [17]; this peak disappeared in the subsequent cycles due to the irreversibility of the SEI layer. The two reduction peaks at ~1.16 V and 1.68 V are attributed to the conversion reactions of SnO_2_ to Sn (Equation (1)) and SnS_2_ to Sn (Equation (2)), respectively [37,38]. The sharp reduction peak at around 0 V is attributed to the reversible insertion of Li-ions into the layers of carbons and formation of Li_x_Sn alloy (Equation (3)). Accordingly, during the first anodic scan, four oxidation peaks located at ~2.38 V, 1.92 V, 1.34 V, and 0.55 V appeared clearly, and the strong peak at 2.38 V is associated with the de-intercalation of Li^+^ ions from the SnS_2_ layers and the decomposition and oxidization of the previously formed SEI [8,39]. The peaks at ~1.92 V and 1.34 V are attributed to the partially reversible conversion reactions of Sn to SnS_2_ and Sn to SnO_2_, respectively [40], and the peak at 0.55 V corresponds to de-intercalation of Li^+^ from Li_x_Sn alloys and carbon layers [17]. In the subsequent cycles, the reduction peaks at ~1.16 V and 1.68 V shift to the positions at about 1.0 V and 1.5 V, meaning the transformation of SnO_2_ or SnS_2_ to metallic Sn is more likely to happen. Meanwhile, the oxidation peak at 2.38 V is diminished obviously, implying the SEI film becomes stable and hardly decomposed. Other than that, the shapes of the other peaks are nearly identical, indicating that electrochemical reaction is highly reversible.

Notably, the initial discharge curve of SnO_2_/SnS_2_@PC appears as a distinct platform at ~1.75 V (Figure 5a), which roughly coincides with the average value of the reduction peak potential (1.68 V) and oxidation peak potential (1.92 V) (Figure 5b). In contrast, both the charge and discharge curves of Pure-C electrode are oblique, and no platforms are observed, implying its energy storage mechanism is physical adsorption without any REDOX reactions. For the SnO_2_/SnS_2_@C electrode, a sloping plateau ranging from ~1 to 1.5 V appeared in the charging curve, whereas, in the discharge curve, the sloping plateau occurs approximately between ~0.5 and 0.7 V. The possible reason is that the carbon in SnO_2_/SnS_2_@C is non-porous, and some SnO_2_ and SnS_2_ nanoparticles were encapsulated by non-porous carbon and thus could not participate in the electrochemical reaction. The cyclability performances of the three samples at a current density of 0.2 A·g^−1^ are displayed in Figure 5c. As shown, with regarded to SnO_2_/SnS_2_@PC, the discharge capacity drops from 1431 mAh·g^−1^ in the first circle to 889 mAh·g^−1^ in the second circle, then gradually decreases to ~715 mAh·g^−1^ at the 20th cycle, and then has a slight increase, and keeps steady at 722 mAh·g^−1^ in the 100th cycle. For the SnO_2_/SnS_2_@C, the cycle discharge curve shows a similar trend as SnO_2_/SnS_2_@PC, but it only delivers an initial capacity of 905 mAh·g^−1^, then drops to 598 mAh·g^−1^ in the second circle and remains at 448 mAh·g^−1^ in the 100th circle. In contrast, for Pure-C, the discharge capacity falls from 441 mAh·g^−1^ in the first circle to 240 mAh·g^−1^ in the second circle and then remains stable until the 100th cycle, exhibiting the best cycle stability among the three samples. This remarkable stability is attributed to the energy storage mechanism of the double-layer capacitor. Different from this type of energy storage mechanism, the SnO_2_/SnS_2_@PC exhibits a hybrid energy storage manner of pseudo-capacitance and double-layer capacitor capacitance, which significantly enhances its capacity. Moreover, the largest surface area and highest pore volume of SnO_2_/SnS_2_@PC is conducive to uphold the structural strain during repeated lithiation/de-lithiation; that is, the porous carbon can effectively provide a buffer space for the volume expansion of SnO_2_ and SnS_2_ particles, enabling them to maintain good structural stability during the cyclic charging/discharging process, thereby exhibiting excellent durability and electrochemical activity compared to the other two materials. Figure 5d shows the rate performance of the SnO_2_/SnS_2_@PC anode at different current rates ranging from 0.2 to 5 A·g^−1^. The average specific capacities are 818, 443, 346, and 245 mAh·g^−1^ at various current densities of 0.2, 0.5, 1.0, and 5.0 A·g^−1^, respectively. When the current density again returned to 0.2 A·g^−1^, the capacity restored to 795 mAh·g^−1^, showing decent reversibility and stability.

**Figure 6:**
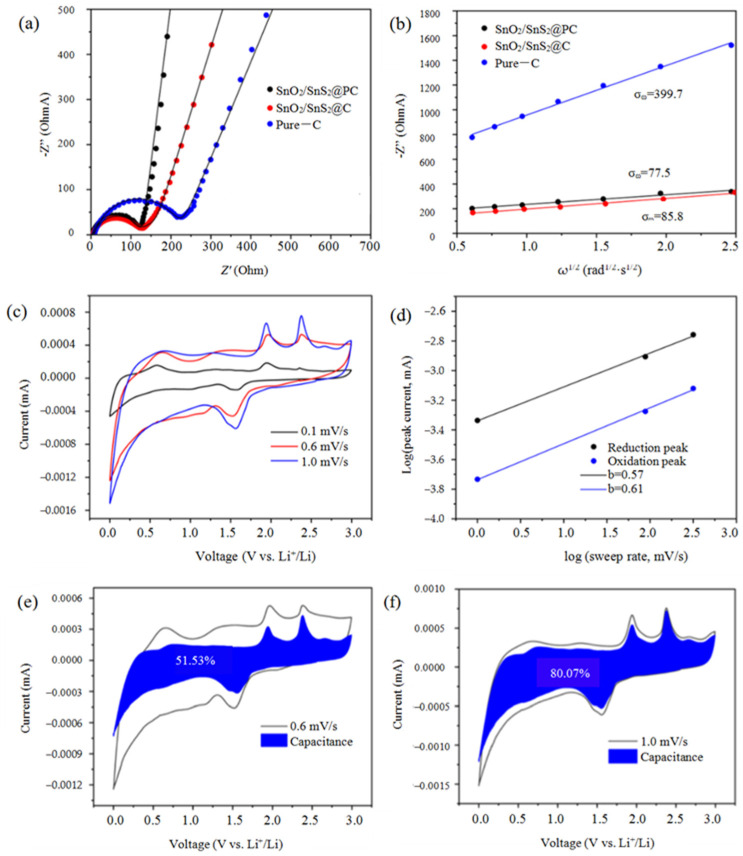
(**a**) Nyquist diagram and corresponding fitting plots; (**b**) fitted curves of Z′ vs. ω^1/2^ at low frequency range; (**c**) CV curves at different scanning rate; (**d**) plots of log(*i*) vs. log(*ν*); (**e**,**f**) capacitance contribution percentages in CV profit at 0.6 and 1.0 mv·s^−1^ for SnO_2_/SnS_2_@PC.

To analyze and quantify the complex electrochemical processes occurring at the electrode/electrolyte interface and evaluate the intrinsic properties of electrode materials, the Nyquist plots of Pure-C, SnO_2_/SnS_2_@C and SnO_2_/SnS_2_@PC anodes were measured and the results are shown in Figure 6a. It is obvious that the solution resistance (*Rs*) obtained from the intercept of the horizontal axis of a semicircle are 1.5, 2.1, and 6.3 Ω, and the charge transfer resistance (*R*_ct_) acquired from the semicircle diameter in the high-frequency region are 121.7 Ω, 134.6 Ω, and 222.8 Ω for SnO_2_/SnS_2_@PC, SnO_2_/SnS_2_@C, and Pure-C, respectively. Both the lowest *Rs* and *R*_ct_ values of SnO_2_/SnS_2_@PC signify its improved conductivity comparing to Pure-C and SnO_2_/SnS_2_@C. Furthermore, in the low frequency range, there are three straight lines with different slopes, the straight line of SnO_2_/SnS_2_@PC is nearly vertical and shows the largest slope, followed by the SnO_2_/SnS_2_@C composite’s slope, and the Pure-C sample’s slope is the smallest. The largest slope of SnO_2_/SnS_2_@PC indicates that it shows the most outstanding double-layer capacitor characteristics, and the electrode process is more characterized by capacitor control behavior, resulting in the greatest enhanced charge-storage performance. Conversely, the electrode process of Pure-C is controlled by diffusion behavior to a greater extent than the other two materials. The rate of Li^+^ diffusion/electron permeation during the electrochemical reactions is further explored though the expression of the lithium-ion diffusion coefficients (*D*_Li_^+^), which can be derived from the following Equation (4) [12,41]:

*D*_Li_^+^ = *R*^2^*T*^2^/2*A*^2^*n*^4^*F*^4^*C*^2^σ^2^ (4)

where *R* is the gas constant, *T* is the absolute temperature, *A* is the surface area of the electrode, *n* is the number of electrons per molecule, *F* is the Faraday constant, *C* is the concentration of Li^+^ ions, and σ is the Warburg coefficient. The δ is obtained according to Equation (5):

*Z*′ = *R*_s_ + *R*_ct_ + σω^1/2^ (5)

where ω is the angular frequency in the lower frequency region, and σ is the slope of the inverse square root plot of Z′ versus the lower angular frequency (ω^1/2^). The linear fit of *Z*’ versus ω^1/2^ is shown in Figure 6b. According to the straight slope σ, the *D*_Li_^+^ valves are calculated as 1.27 × 10^−11^, 9.74 × 10^−13^, 3.66 × 10^−14^ for SnO_2_/SnS_2_@PC, SnO_2_/SnS_2_@C, and Pure-C, respectively. Obviously, the SnO_2/_SnS_2_@PC possess the largest *D*_Li_^+^, indicating that it achieves the fastest charge transfer and thus greatly improves the lithium storage performance. These fine properties attribute to two facts, one is that the high specific surface area of the porous material provides more surface redox active sites, and the other is that the open pore structure facilitates the rapid transport ion.

The electrochemical reaction kinetics was further investigated through record of the CV curves of the SnO_2_/SnS_2_@PC anode at different scan rates of 0.2, 0.6, and 1.0 mV/s. As shown in Figure 6c, the intensity of the redox peak current increases with the increasing of scan rate. In general, the relationship between the intensity of the peak current (*i*) and the scan rate (*ν*) can be expressed as Equations (6) and (7) [40,41].

*i* = *aν*^*b*^ (6)

log(*i*) = *b*log(*ν*) + log(*a*) (7)

where *a* and *b* are empirical parameters. The magnitude of the *b* value directly reflects the control steps for charge storage, which usually includes battery type (diffusion control) behavior and capacitive behavior (surface control). While *b* = 0.5, the electrode process is controlled by diffusion, and the reaction rate is mainly determined by the diffusion speed of ions in the bulk phase of the electrode material, manifesting as typical battery-type behavior. While *b* = 1.0, the electrode process is a typical capacitive behavior and is surface controlled, meaning that the reaction rate depends on the rapid process occurring at the electrode/electrolyte interface, and ion diffusion is unrestricted [24]. As seen in Figure 6b, the *b* values for reduction reaction and oxidation reaction are 0.57 and 0.61 respectively; both are intermediate between 0.5 and 1, implying a mixed charge storage process, i.e., capacitive (surface-based) and diffusion-based processes. The contributions of the above two processes can be further quantified through the following methods. According to Dunn’s method, the overall accumulated charge at the SnO_2_/SnS_2_@ PC electrode can be split into two parts as in the following Equation (8) [14,21]:

*i*/*ν*^1/2^ = *k*_1_*ν*^1/2^ + *k*_2_ (8)

where *k*_1_*ν*^1/2^ and *k*_2_ are denoted as the contributions of capacitance (includes double-layer capacitance and pseudo-capacitance) and the diffusion-controlled reaction processes, respectively. Drawing a line by *i*/*ν*^1/2^ versus *k*_1_*ν*^1/2^, the *k*_1_ and *k*_2_ can be determined from the slope and intercept of the line, and hence, the capacitance contribution and diffusion contribution can be obtained. Figure 6e,f show the capacitance contribution percentage in the CV profile at a scan rate of 0.6 and 1.0 mv·s^−1^, and the capacitance contribution are found to be 51.53% and 80.07%, respectively, indicating that the capacitive-controlled process has a higher proportion than the diffusion-based process, and the higher the scan rate is, the greater the capacitance contribution fraction will be, which is conducive to the Li ion storage kinetics and delivers excellent rate performance of the material [42].

## 4. Conclusions

In summary, we have successfully synthesized a porous SnO_2_/SnS_2_@PC anode material for LIBs by calcinating a mixture of SnO_2_ nanoparticles, petroleum asphalt, and spherical CaCO_3_ at high temperature under a nitrogen atmosphere. By the decomposition of CaCO_3_ at high temperature, the porous carbon with a three-dimensional structure was obtained. The key novelty of this work lies in fully utilizing the abundant sulfur element in petroleum asphalt, and in situ generating SnS_2_ through high-temperature carbonization of a mixture of SnO_2_, petroleum asphalt, and calcium carbonate, which greatly reduces the emission of the harmful element sulfur into the atmosphere. The generated SnS_2_ and SnO2 are uniformly combined and distributed in a single conductive porous carbon matrix; thus, the SnS_2_/SnO_2_/PC anode material with good structural stability and significantly improved electrochemical performance was constructed. The performance of this in situ constructed SnS_2_/SnO_2_/PC anode material is far superior to that of mixture obtained by physical mixing of the SnO_2_, SnS_2_, and PC used in the relevant literature.

The as-prepared SnO_2_/SnS_2_@PC composite exhibits a specific surface area of 190 m^2^·g^−1^ with total pore volume 0.386 cm^3^·g^−1^, which is nearly 82 times higher than that of Pure-C and SnO_2_/SnS_2_@C without void structures. As a potential substitute for graphite, an anode material of LIBs, the SnO_2_/SnS_2_@PC anode delivers an initial capacity of 1431 mAh·g^−1^ with a Coulomb efficiency of 74.6% at a current density of 0.2 A·g^−1^ and remains at a steady value of ~720 mAh·g^−1^ over the 20th to 100th cycles, showing good charge/discharge cycle stability. The remarkable improvement in capacity compared with Pure-C is attributable to the combined contributions of pseudo-capacitance and double-layer capacitance with the pseudo-capacitance contribution being predominant. Since the porous carbon provides sufficient buffer space for the expansion of SnO_2_ and SnS_2_ particles, the SnO_2_/SnS_2_@ PC composite exhibits good structural stability during the charging/discharging process. This work provides a low-cost and simple method for production of Sn-base/carbon anode materials with high capacity and stability, and hence has a potential application prospect.

## Figures and Tables

**Table 1:** BET and BJH data of Pure-C, SnO_2_@SnS_2_@C, and SnO_2_@SnS_2_@PC.

Samples	S_Total_ (m^2^·g^−1^)	S_Mic_ (m^2^·g^−1^)	S_Mec_ (m^2^·g^−1^)	V_Mac_ (cm^3^·g^−1^)	V_Mec_ (cm^3^·g^−1^)	V_Total_ (cm^3^·g^−1^)
Pure-C	2.3	0.1	2.2	0	0.001	0.001
SnO_2_/SnS_2_@C	3	0.4	2.6	0	0.005	0.005
SnO_2_/SnS_2_@PC	190	17	173	0.006	0.380	0.386

## Data Availability

The original contributions presented in this study are included in the article. Further inquiries can be directed to the corresponding author.
